# The relationship between college students' AI technology dependence and learning burnout: a chain mediation analysis of technology acceptance and AI self-efficacy

**DOI:** 10.3389/fpsyg.2026.1843366

**Published:** 2026-05-22

**Authors:** Chen Gu, Bingchen He, Xinping Zhang

**Affiliations:** 1 School of Education Science, Nanjing Normal University, Nanjing, China; 2 School of Undergraduate Studies, Nanjing University of Posts and Telecommunications, Nanjing, China; 3 Faculty of Education, The University of Melbourne, Melbourne, VIC, Australia

**Keywords:** AI self-efficacy, AI technology dependence, college students, learning burnout, technology acceptance

## Abstract

**Objective:**

Against the backdrop of the deep integration of generative artificial intelligence into higher education, the non-adaptive use of AI learning tools among college students has become a critical risk factor affecting learning psychology and academic adaptation.

**Methods:**

Based on the Technology Acceptance Model (TAM) and Self-Efficacy Theory (SET), this study constructed a chain mediation model to examine the sequential mediating associations of technology acceptance and AI self-efficacy in the relationship between AI technology dependence and learning burnout.

**Results:**

The results show that AI technology dependence is significantly positively associated with learning burnout, technology acceptance plays a significant independent mediating role between AI technology dependence and learning burnout, AI self-efficacy plays a significant independent mediating role between AI technology dependence and learning burnout, technology acceptance and AI self-efficacy form a significant chain mediation path.

**Conclusion:**

This study clarifies the multi-level psychological transmission mechanism of AI technology dependence is related to learning burnout, enriches theoretical achievements in the fields of AI education application and learning burnout, and provides empirical basis and practical implications for universities to carry out AI literacy education, alleviate college students' learning burnout, and promote a healthy and sustainable human-computer collaborative learning model.

## 1 Introduction

With the rapid iteration and application of generative artificial intelligence (GenAI) in educational scenarios worldwide, AI learning tools represented by ChatGPT, Gemini, intelligent Q&A systems, and academic auxiliary tools have been deeply embedded in college students' learning processes such as classroom learning, after-class assignments, and literature research (Elhag et al., 2025; Fardian et al., 2025). GenAI has significantly improved learning efficiency and flexibility with its powerful capabilities in natural language processing, information integration, real-time feedback, and personalized content generation, becoming an indispensable support under the new normal of digital learning (Hu and Shao, 2025; Zhang et al., 2025). However, along with technological empowerment, non-adaptive technology use behaviors such as overuse, cognitive offloading, passive task completion, and emotional attachment have become increasingly common, gradually forming a new type of learning problem centered on AI technology dependence (Chirayath et al., 2025; Tian and Zhang, 2025). Such dependence is not only reflected in excessive use frequency and generalized scenarios, but also accompanied by deep psychological changes such as reduced independent thinking, decreased metacognitive participation, and lower sense of academic accomplishment, exerting potential negative impacts on students' learning motivation, emotional experience, and academic adaptation (Yavich, 2025).

Learning burnout is a persistent negative psychological state formed by college students under long-term academic pressure, repetitive learning experiences, and low sense of accomplishment, which is a core indicator predicting academic withdrawal, academic procrastination, mental health problems, and even academic interruption (Huang et al., 2025; Yang and Tu, 2025). In recent years, an increasing number of studies have focused on the relationship between digital technology use and learning burnout, and most studies have confirmed a significant positive correlation between excessive technology use, digital addiction, and learning burnout (Lu et al., 2025; Zhang and Liu, 2025). However, in the specific context of AI learning, relevant research still has obvious deficiencies: first, most studies regard AI use as an overall variable and fail to distinguish between reasonable empowering use and non-adaptive dependence. Second, few studies reveal the complete psychological linkage pattern of how AI technology dependence is related to learning burnout. Third, few studies include both technology acceptance and AI self-efficacy into the model, making it difficult to explain how external technology behaviors affect learning psychological outcomes through internal cognition and efficacy beliefs (Shi et al., 2026; Wang and Xu, 2026).

Based on the above realistic background and theoretical gaps, this study takes the Technology Acceptance Model and Self-Efficacy Theory as dual theoretical supports, takes technology acceptance as the core variable of external technology cognition and AI self-efficacy as the core variable of domain-specific ability beliefs, and constructs a chain mediation model of “AI technology dependence → technology acceptance → AI self-efficacy → learning burnout.” Thus, this study aims to improve the theoretical explanation framework of learning psychology in the AI environment and provide operable practical paths for universities to carry out AI literacy education, prevent and alleviate learning burnout, and build a healthy human-computer collaborative learning ecosystem.

## 2 Literature review and research hypotheses

### 2.1 Theoretical framework

The Technology Acceptance Model (TAM) is one of the most influential theories explaining individual technology use behaviors (Davis, 1989; King and He, 2006). This model holds that an individual's acceptance of technology is mainly determined by perceived usefulness and perceived ease of use, which are further related to attitude, intention, and actual use behavior (Davis, 1989; Granić and Marangunić, 2019). Rational and positive technology acceptance can promote effective, controllable, and sustainable technology use, while passive, forced, or irrational acceptance easily leads to overuse, dependence, and even negative consequences (Ariff et al., 2025). In the AI learning context, technology acceptance reflects students' stable, healthy, and rational cognitive evaluation of AI tools and is a key cognitive bridge connecting external technology use behaviors and internal learning psychological changes (Yan et al., 2024). In recent years, a large number of AI education studies based on TAM have further enriched the application scenarios and connotations of the theory (Boughanzai et al., 2026; Ning et al., 2026).

Self-Efficacy Theory (SET), proposed by Bandura, holds that an individual's efficacy beliefs in a specific field directly affect their motivation, emotional state, persistence, and behavioral performance (Bandura et al., 1980). AI self-efficacy is a domain-specific efficacy belief, referring to students' confidence and judgment in effectively, reasonably, and independently using AI tools to complete learning tasks (Wang and Chuang, 2024). Students with high AI self-efficacy are more likely to actively regulate their use behaviors, maintain stable emotions when facing difficulties, and show less anxiety and avoidance. Students with low AI self-efficacy are more likely to rely on external tools, avoid complex tasks, and have emotional fluctuations when use is blocked (Zhang et al., 2024). Many studies have confirmed that AI self-efficacy is a key variable affecting college students' AI use behaviors and learning psychology. Klarin et al. (2024) found that AI self-efficacy is significantly positively associated with students' academic performance and negatively associated with learning burnout, serving as an important protective factor to buffer the negative impact of technology dependence and protect students' mental health.

Thus, the TAM and SET can be integrated into a cohesive hierarchical framework that explains how maladaptive AI use relates to learning burnout. Technology acceptance, as conceptualized in TAM, represents a relatively external perceptual cognitive level of psychological response. It reflects students evaluative judgments toward the utility and experience of using AI tools, which also acts as the primary cognitive interface between technology use and internal psychological states. By contrast, AI self-efficacy, rooted in SET, represents a deeper competence based belief level. This belief level emerges from sustained cognitive evaluation and successful usage experience. Theoretically, this sequential ordering is not arbitrary. Students perceptual and evaluative responses to AI provide the foundational cognitive context for the development of their confidence in using AI. Therefore, rather than operating as parallel mediators, technology acceptance and AI self-efficacy form a logically consistent chain mediation pathway. In this pathway, external cognitive appraisal precedes and shapes domain specific efficacy beliefs. This integrated sequential structure enables a comprehensive explanation of how AI dependence gradually translates into disrupted learning psychology.

### 2.2 The relationship between AI technology dependence and learning burnout

AI technology dependence refers to college students' excessive and compulsive use of AI tools in learning activities, outsourcing a large number of cognitive tasks, reducing independent thinking, in-depth processing and metacognitive regulation, and showing non-adaptive behavioral tendencies such as anxiety, procrastination, and learning stagnation when unable to use AI (Huang S. et al., 2024). Its core characteristics are reflected in three aspects: first, cognitive offloading, that is, actively handing over cognitive tasks such as memory, thinking, and reasoning that should be completed independently to AI, reducing their own in-depth cognitive participation (Jose et al., 2025; Qamar et al., 2025). Second, tool dependence, that is, over-relying on the output results of AI tools, lacking critical thinking and verification of the results, and even completely copying the answers given by AI (Dergaa et al., 2024). Third, emotional attachment, that is, forming a strong psychological dependence on AI tools, regarding AI as an indispensable technical support in the learning process, and showing obvious anxiety, unease and decreased learning ability when unable to use AI (Chen C. et al., 2025).

Learning burnout is a persistent negative psychological state formed by college students under long-term academic pressure and negative learning experiences, mainly including three dimensions: low mood, inappropriate behavior, and low sense of accomplishment (Liu et al., 2025; Maslach, 2003). In recent years, many empirical studies have consistently shown that excessive AI use and technology dependence are positively associated with college students' learning burnout, with a stable positive correlation between the two. Zhang et al. (2024) found that the higher the AI dependence, the more obvious the cognitive offloading behavior, the lower the learning motivation and engagement, the more significant the emotional exhaustion and inappropriate behavior symptoms, and the higher the learning burnout level. Dergaa et al. (2024) showed that long-term reliance on AI to complete learning tasks will significantly reduce students' sense of autonomy, learning engagement and academic accomplishment, and students gradually lose the ability to think and solve problems independently, and then fall into a vicious circle of “dependence-inefficiency-frustration-burnout,” and finally show obvious learning burnout symptoms. Huang et al. (2025) further confirmed that AI technology dependence is significantly positively associated with college students' learning burnout level in subsequent semesters, and this predictive effect is stable. Based on this, this study proposes:

**H1: College students' AI technology dependence is significantly positively associated with learning burnout**.

### 2.3 The mediating role of technology acceptance

Technology acceptance is a comprehensive reflection of an individual's perceived usefulness, perceived ease of use and positive use intention of AI learning tools, representing an individual's rational and healthy cognition and attitude toward AI tools (Huang F. et al., 2024; Schiavo et al., 2024). In the AI learning context, students with high technology acceptance can clearly recognize the advantages and limitations of AI tools, regard AI as a learning aid, reasonably regulate their use behaviors, and take the initiative to use AI tools to improve learning efficiency and quality. Students with low technology acceptance often have biased cognition of AI tools, either over-relying on AI tools as omnipotent, or rejecting AI tools, unable to give full play to the positive role of AI tools (Huang S. et al., 2024; Zhang et al., 2024).

Existing empirical studies have consistently confirmed a significant negative correlation between AI technology dependence and technology acceptance. Donmez and Seferoglu (2025) pointed out that excessive and non-adaptive AI dependence reduces students' perceived usefulness and ease of use of technology, making it difficult for them to form a stable and rational technology acceptance attitude, and then showing passive and compulsive use characteristics. Ning et al. (2026) further verified that problematic AI use is significantly negatively correlated with perceived usefulness and ease of use, indicating that the higher the dependence, the more negative students' recognition and experience of technology value, and the lower the technology acceptance level. At the same time, technology acceptance has a significant negative predictive effect on learning burnout and is a key protective factor to alleviate burnout. This research also showed that students with higher technology acceptance have a stronger sense of autonomy and competence in AI-assisted learning, more stable emotions, and lower learning burnout level (Ning et al., 2026). Li?an (2025) found that positive and rational technology acceptance can effectively buffer technostress, reduce emotional exhaustion and inappropriate behaviors, and thus significantly alleviate learning burnout. Based on the above research conclusions, it can be clearly inferred that AI technology dependence is positively associated with learning burnout by reducing individuals' technology acceptance level, and technology acceptance plays a significant mediating role between them. Based on this, this study proposes:

**H2: Technology acceptance plays a significant mediating role between AI technology dependence and learning burnout**.

### 2.4 The mediating role of AI self-efficacy

AI self-efficacy refers to college students' confidence and judgment in effectively, reasonably and independently using AI learning tools to complete learning tasks and solve learning problems, which is a specific embodiment of self-efficacy in the field of AI learning (Wang and Chuang, 2024). It reflects students' cognition and evaluation of their own AI use ability, and is a key variable affecting students' AI use behaviors and learning psychology. Many latest empirical studies show that AI technology dependence will significantly weaken college students' AI self-efficacy. Zhang et al. (2024) found that students with high AI dependence have significantly lower self-efficacy than those with low AI dependence, because they rely on AI tool output results for a long time and lack successful experience obtained through independent regulation and exploration, making it difficult to form a positive cognition of their own AI use ability. Klarin et al. (2024) pointed out that long-term use of AI to replace independent thinking will reduce students' perception of their own abilities, and gradually make students lose confidence in solving problems independently, thereby reducing AI self-efficacy.

Moreover, AI self-efficacy is significantly negatively associated with learning burnout and is a core protective factor to buffer learning burnout. Wang and Xu (2026) confirmed that the higher the self-efficacy, the more positive students' emotional experience in human-computer collaborative learning, the better they can cope with learning difficulties and AI use obstacles, reduce loneliness and inappropriate behaviors, and thus reduce learning burnout level. Kim and Kim (2024) found that self-efficacy can directly reduce emotional exhaustion, reduce inappropriate behaviors and improve sense of accomplishment in the AI learning environment, and is an important protective factor against learning burnout. Thus, it can be clearly inferred that AI technology dependence will be positively associated with learning burnout by reducing individuals' AI self-efficacy, and AI self-efficacy plays a significant mediating role. Based on this, this study proposes:

**H3: AI self-efficacy plays a significant mediating role between AI technology dependence and learning burnout**.

### 2.5 The chain mediating role of technology acceptance and AI self-efficacy

On the basis of integrating the two theories and all variable relationships, this study further proposes a complete chain mediation hypothesis. According to the transmission logic of external technology behavior, internal technology cognition, domain-specific efficacy beliefs and learning psychological outcomes, as a non-adaptive use behavior, AI technology dependence reduces students' rational cognition and positive evaluation of AI tools, leading to a decline in technology acceptance. The decline in technology acceptance means that students cannot obtain stable and effective support experience from AI use, and then weaken AI self-efficacy. Finally, under the condition of dual psychological resource damage of technology acceptance and AI self-efficacy, learning burnout is significantly associated.

**Figure 1:**
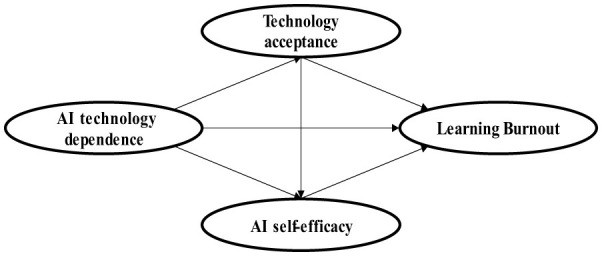
Study hypothetical model diagram.

This chain path is strongly supported by both the TAM and SET, and many studies have indirectly verified its rationality. The above studies have confirmed that technology acceptance is significantly positively associated with AI self-efficacy, AI self-efficacy is significantly negatively associated with learning burnout, and AI technology dependence affects AI self-efficacy by reducing technology acceptance, and finally increases learning burnout, indirectly verifying the existence of the chain mediation path. Based on this, this study proposes:

**H4: Technology acceptance and AI self-efficacy play a significant chain mediating role between AI technology dependence and learning burnout (as shown in** **Figure 1****)**.

## 3 Methods

### 3.1 Participants

This study adopted the convenient sampling method to conduct an online questionnaire survey on full-time undergraduates from five ordinary universities in China through the Wenjuanxing platform from September to October 2025, obtaining 508 data. Before the survey, the research purpose, filling standards and data confidentiality principles were clearly explained to the subjects, and the subjects completed the questionnaire on the premise of informed consent and voluntary participation, and all procedures met ethical requirements. The inclusion criteria of the study subjects were having used at least one AI learning tool (such as ChatGPT, Gemini, Doubao, etc.) and being able to answer completely and carefully. After excluding invalid questionnaires such as too short filling time, regular answers and missing key items, 466 valid questionnaires were finally obtained, with an effective recovery rate of 91.73%. Among the valid samples, 339 were males (72.75%) and 127 were females (27.25%).

### 3.2 Research measures

#### 3.2.1 AI technology dependence

AI technology dependence was measured by the conversational AI dependence scale of Chen Y. et al. (2025). The scale includes four dimensions and 20 items: Uncontrollability, Withdrawal symptoms, Mood modification, and Negative impact. The scale uses a five-point scoring method (1 = completely inconsistent, 5 = completely consistent), and the higher the total score, the higher the AI technology dependence. In this study, the Cronbach's α coefficient of the total scale is 0.735, the α coefficients of each dimension are 0.715, 0.796, 0.776, and 0.702.

#### 3.2.2 Technology acceptance

Technology acceptance was measured by the accept artificial intelligence-driven technology scale of Molefi et al. (2024). The scale includes 6 dimensions and 27 items: Perceived Usefulness of AI-driven technology, Perceived Ease of Use of AI-driven technology, Technical Proficiency in AI-driven technology, School Support and Resources for AI-driven technology, Attitude toward AI-driven technology, and Behavioral intention to use AI-driven technology. According to the research needs, this study only adopted two dimensions of perceived usefulness and perceived ease of use, a total of 10 items. The scale uses a five-point scoring method (1 = strongly disagree, 5 = strongly agree), and the higher the total score, the higher the rational acceptance of AI learning tools. In this study, the Cronbach's α coefficient of the total scale is 0.905, the α coefficients of each dimension are 0.863, and 0.844.

#### 3.2.3 AI self-efficacy

AI self-efficacy was measured by the AI self-efficacy scale of Wang and Chuang (2024). The scale includes four dimensions and 22 items: Assistance, Anthropomorphic Interaction, Comfort with AI, and Technological skills. The scale uses a five-point scoring method (1 = completely inconsistent, 5 = completely consistent) to measure students' confidence and ability judgment in effectively and reasonably using AI to complete learning tasks. The higher the total score, the stronger the AI self-efficacy. In this study, the Cronbach's α coefficient of the total scale is 0.977, the α coefficients of each dimension are 0.919, 0.933, 0.904, and 0.914.

#### 3.2.4 Learning burnout

Learning burnout was measured by the College Students' Learning Burnout Scale compiled by Lian et al. (2005) and widely cited in international research. The scale includes three dimensions and 20 items: Low mood, Inappropriate behavior, and Low sense of accomplishment. The scale uses a five-point scoring method (1 = completely inconsistent, 5 = completely consistent), and the higher the total score, the more serious the learning burnout. In this study, the Cronbach's α coefficient of the total scale is 0.914, the α coefficients of each dimension are 0.908, 0.836, and 0.891.

### 3.3 Data analysis

After all data were entered, cleaned and outliers processed, descriptive statistics, Pearson correlation analysis and Harman's single-factor common method bias test were performed using SPSS 26.0. AMOS 27.0 was used to construct a structural equation model to test the fitting of the hypothetical model. The bias-corrected bootstrap (BC) method with 5,000 resamples was used to test the mediating and chain mediating effects. The 95% bias-corrected confidence intervals (95% BC CIs) are reported. Effects were considered significant if the intervals did not include 0.

## 4 Results

### 4.1 Common method bias test

All data in this study were self-reported by the subjects, and diagnosed by Harman's single-factor test. The unrotated exploratory factor analysis of all scale items showed that 11 common factors with characteristic roots greater than 1 were extracted, among which the variance explanation rate of the first common factor was 39.316%, less than the critical standard of 40%, indicating that there is no serious common method bias in this study, and the data results are reliable (Podsakoff et al., 2003).

### 4.2 Descriptive statistics and correlation analysis

This study conducted descriptive statistics and Pearson correlation analysis on the four core variables of AI technology dependence, technology acceptance, AI self-efficacy, and learning burnout, as shown in Table 1. The results showed that the mean value of AI technology dependence was 2.644, standard deviation was 0.409, skewness was −0.714, kurtosis was 0.533. The mean value of technology acceptance was 3.787, standard deviation was 0.619, skewness was 0.113, and kurtosis was 0.371. The mean value of AI self-efficacy was 4.028, standard deviation was 0.615, skewness was −0.294, and kurtosis was 0.625. The mean value of learning burnout was 2.150, standard deviation was 0.616, skewness was −0.086, kurtosis was −0.419. The absolute values of skewness and kurtosis of all variables are within the acceptable range, the data basically conform to the normal distribution assumption, and are suitable for subsequent statistical analysis. Correlation analysis showed that AI technology dependence was significantly negatively correlated with technology acceptance (*r* = −0.362, *p* < 0.001), significantly negatively correlated with AI self-efficacy (*r* = −0.334, *p* < 0.001), and significantly positively correlated with learning burnout (*r* = 0.241, *p* < 0.001). Technology acceptance was significantly positively correlated with AI self-efficacy (*r* = 0.713, *p* < 0.001), and significantly negatively correlated with learning burnout (*r* = −0.597, *p* < 0.001). AI self-efficacy was significantly negatively correlated with learning burnout (*r* = −0.622, *p* < 0.001). The correlation direction between variables is completely consistent with the theoretical hypothesis of this study, providing necessary statistical support for the subsequent structural equation model test and chain mediating effect analysis.

**Table 1:** Descriptive statistics and correlation analysis of variables (*N* = 466).

Variables	Mean	SD	Skewness	Kurtosis	1	2	3	4
1. AI technology dependence	2.644	0.409	−0.714	0.533	1			
2. technology acceptance	3.787	0.619	0.113	0.371	−0.362^**^	1		
3. AI self-efficacy	4.028	0.615	−0.294	0.625	−0.334^**^	0.713^**^	1	
4. learning burnout	2.150	0.616	−0.086	−0.419	0.241^**^	−0.597^**^	−0.622^**^	1

^**^p < 0.01.

### 4.3 Mediation effect test

The Bootstrap method was used to test the chain mediation model, and the results are shown in Table 2. The 95% confidence intervals of the independent mediating effect of technology acceptance, the independent mediating effect of AI self-efficacy, and the chain mediating effect of technology acceptance and AI self-efficacy do not contain 0, indicating that the three types of indirect effects are significant. The total indirect effect accounts for more than 60% of the total effect, indicating that most of the relationship of AI technology dependence on learning burnout is realized through indirect paths. The chain mediating path effect is significant, indicating that AI technology dependence eventually is positively associated with learning burnout through the continuous process of reducing technology acceptance to weakening AI self-efficacy, and all hypotheses of this study are supported.

**Table 2:** Test results of chain mediation effect (bootstrap = 5,000).

Path	Effect size	Standard error	95%CI	
			Lower limit	Upper limit
Path1	0.171	0.038	0.099	0.247
Path2	0.053	0.021	0.014	0.096
Path3	0.149	0.028	0.096	0.205
Total indirect effect	0.373	0.050	0.271	0.469
Direct effect	0.010	0.057	0.102	0.122
Total effect	0.383	0.068	0.230	0.497

Path 1: AI technology dependence → technology acceptance → learning burnout. Path 2: AI technology dependence → AI self-efficacy → learning burnout. Path 3: AI technology dependence → technology acceptance → AI self-efficacy → learning burnout.

**Figure 2:**
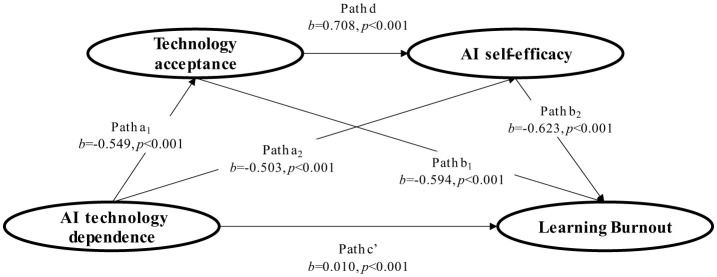
Path coefficient diagram of chain intermediary model.

Taking AI technology dependence as the independent variable, learning burnout as the dependent variable, and technology acceptance and AI self-efficacy as chain mediating variables, a structural equation model was constructed. The proposed theoretical model and standardized path coefficients are presented in Figure 2. Path: Represents the influence path. a_1_: The regression coefficient of AI technology dependence → technology acceptance. a_2_: The regression coefficient of AI technology dependence → AI self-efficacy. d: The regression coefficient of technology acceptance → AI self-efficacy. b_1_: The partial regression coefficient of technology acceptance → learning burnout. b_2_: The partial regression coefficient of AI self-efficacy → learning burnout. c': The direct effect of AI technology dependence → learning burnout after incorporating technology acceptance and AI self-efficacy. *b*: The estimated value of the regression coefficient corresponding to the path. *p*: The significance test value of the path coefficient. The overall fitting results of the model are: χ^2^/df = 2.314, GFI = 0.927, AGFI = 0.902, NFI = 0.918, CFI = 0.946, TLI = 0.901, RMSEA = 0.054, and SRMR = 0.049, all fitting indexes reach the ideal standard, and the model fits the data well. This result indicated that AI technology dependence shows a significant positive direct association with learning burnout, AI technology dependence is significantly negatively associated with technology acceptance, technology acceptance is significant positive associated with AI self-efficacy; technology acceptance and AI self-efficacy are significant negative associated with learning burnout, respectively. The above results provide support for further testing the mediating effect and chain mediating effect.

## 5 Discussion

Based on the TAM and SET, this study systematically explored the relationship of college students' AI technology dependence on learning burnout and its chain mediating mechanism. The results show that AI technology dependence is not only directly positively associated with learning burnout, but also indirectly related to learning burnout through the independent mediating paths of reducing technology acceptance and weakening AI self-efficacy, as well as the chain mediating path of technology acceptance and AI self-efficacy. This result deepens the understanding of the learning psychological mechanism of college students in the intelligent era, and is highly consistent with the conclusions of mainstream international studies in recent years.

Firstly, the significant positive direct association between AI technology dependence and learning burnout is supported, which is consistent with the Conservation of Resources Theory and the findings of many recent empirical studies (Dergaa et al., 2024; Zhang et al., 2024). Over-reliance on AI tools will make college students reduce in-depth cognitive processing and independent learning investment, stay in a passive learning state for a long time, leading to a decline in learning control, increased emotional exhaustion, and decreased sense of accomplishment, and finally show obvious burnout symptoms. This result is completely consistent with the research conclusion that cognitive offloading weakens learning investment, fully indicating that AI technology dependence is an important risk factor for learning burnout, and universities must be alert to the negative learning consequences caused by non-adaptive technology dependence when promoting AI education applications (Chirayath et al., 2025; Lu et al., 2025).

Secondly, the significant independent mediating roles of technology acceptance and AI self-efficacy reveal the protection mechanism from two levels of technology cognition and ability belief. The higher the AI technology dependence, the more difficult it is for individuals to form rational and stable technology acceptance, cannot use AI as an effective learning resource, and then increase burnout; at the same time, AI dependence will reduce independent successful experience and lower AI self-efficacy, and low self-efficacy is an important antecedent variable of burnout (Wang and Chuang, 2024; Yang and Tu, 2025). This conclusion shows that improving technology acceptance and protecting AI self-efficacy are the key entry points to alleviate the negative impact of AI dependence.

Finally, the core finding of this study is the chain mediating role of technology acceptance and AI self-efficacy. AI technology dependence first damages individuals' rational cognition of AI tools, leading to a decline in technology acceptance. Low-level technology acceptance cannot provide effective learning support experience, further weakening AI self-efficacy. The loss of dual resources jointly promotes the occurrence and development of burnout. This chain path clearly presents the complete transmission chain of external technology use behavior, internal technology cognition, core ability belief, and learning psychological adaptation, which not only integrates the TAM and SET, but also provides a clear path for precise intervention.

## 6 Practical implications

The present findings yield three sets of targeted practical implications for advancing AI-integrated pedagogy, alleviating learning burnout, and fostering sustainable human–computer collaborative learning in higher education.

Firstly, institutions should systematically strengthen AI literacy education to guide adaptive AI utilization and mitigate maladaptive cognitive offloading. Universities may embed AI literacy modules into general education curricula, covering evidence-based AI tool operation, boundaries between AI assistance and independent learning, critical evaluation of generated content, and academic integrity norms. Educators can design scaffolded, task-based activities that encourage students to use AI for information retrieval and preliminary structuring while requiring independent reasoning, original argumentation, and manual verification of core content. Complementary workshops and peer-sharing sessions can further clarify the risks of over-reliance and passive dependency, helping students internalize responsible usage norms.

Secondly, stakeholders should enhance students' rational technology acceptance by strengthening perceived usefulness and perceived ease of use, thereby consolidating the belief that AI serves as a supportive supplement rather than a replacement for independent learning. Higher education institutions may optimize the deployment of user-friendly AI learning platforms and deliver structured skill-training sessions to lower operational barriers. In disciplinary teaching, instructors may explicitly highlight the complementary value of AI in boosting efficiency without compromising deep learning, helping students form balanced perceptions of technological affordances. Such improvements in rational acceptance can reduce compulsive usage and emotional over-reliance, thereby buffering against learning burnout.

Thirdly, educators should protect and enhance students' AI self-efficacy via process-oriented assessment, incremental success experiences, and metacognitive scaffolding to interrupt the burnout transmission chain. Universities may adopt formative evaluation that rewards effort, progress, and self-regulated AI usage rather than focusing solely on final outcomes. Educators can design tiered learning tasks aligned with individual proficiency levels to enable repeated mastery experiences, which strengthen efficacy beliefs. Metacognitive reflection protocols may also be implemented to help students monitor their usage patterns, recognize early signs of dependency, and adjust behaviors proactively. Together, these strategies sustain motivational resources and reduce vulnerability to learning burnout.

## 7 Limitations

This study has certain limitations that need to be improved and supplemented in future research.

First, in terms of research design, this study adopts a cross-sectional design, which only collects data at a single time point, making it difficult to infer the causal relationship between variables such as AI technology dependence, technology acceptance, AI self-efficacy, and learning burnout. Although the correlation analysis and structural equation model test show a certain relationship between variables, the dynamic changes of variables and the direction of causal influence cannot be fully reflected.

Second, in terms of sample selection, this study mainly selects undergraduates from five ordinary universities in China through convenient sampling, and the sample scope is relatively limited. The gender ratio of the sample is unbalanced (72.75% males and 27.25% females), and the sample does not cover students of different education levels (such as junior college students, postgraduates), different disciplines (such as liberal arts, science, engineering, and medicine) and different regions (such as rural and urban areas), which affects the representativeness and generalization of the research results.

Third, in terms of variable setting, this study only focuses on the chain mediating role of technology acceptance and AI self-efficacy, and does not consider the role of potential moderating variables. For example, discipline type may be related to students' frequency and way of using AI tools, and then affect the relationship between AI technology dependence and learning burnout; learning motivation (such as intrinsic motivation and extrinsic motivation) may moderate the relationship of AI self-efficacy on learning burnout, but these variables are not included in the research model, which makes the research conclusion lack more comprehensive theoretical support.

Fourth, the present study adopted a cross-sectional design, which limits the ability to draw firm causal conclusions or determine temporal order. Although we proposed a chain mediation model from AI technology dependence to learning burnout, reverse causation cannot be ruled out. It is equally plausible that students with higher learning burnout may be more likely to use or depend on AI tools to cope with academic stress, reduce cognitive effort, or avoid difficult learning tasks, rather than AI dependence leading to burnout. In addition, this study tested only one theoretically derived model without comparing it with alternative model specifications. Future research may consider examining alternative or competing models to further verify the robustness and superiority of the proposed chain mediation structure.

## 8 Future research directions

In view of the above results and limitations, future research can be carried out from the following aspects to further improve the research. First, adopt a longitudinal follow-up research design, select a fixed sample group, and collect data at multiple time points (such as the beginning, middle, and end of the semester) to track the dynamic changes of variables such as AI technology dependence and learning burnout, and more accurately infer the causal relationship between variables. Second, expand the sample scope and optimize the sample structure. In future research, stratified sampling can be adopted to select students from different education levels, disciplines, genders, and regions, so as to improve the representativeness of the sample and enhance the generalization of the research results. Third, introduce moderating variables to enrich the research model. Future research can explore the moderating role of variables such as discipline type, learning motivation, AI literacy and teacher guidance in the relationship between AI technology dependence and learning burnout, so as to further clarify the applicable boundary and stability of the chain mediating model. In addition, future research can also combine qualitative and quantitative research methods, such as conducting in-depth interviews with students and teachers, to deeply explore the specific psychological mechanism of AI technology dependence affecting learning burnout, and provide more targeted practical enlightenment for AI literacy education in universities.

## Data Availability

The raw data supporting the conclusions of this article will be made available by the authors, without undue reservation.
